# Case report: Mixed infection of bovine papillomaviruses associated with squamous papilloma of the upper alimentary tract in a dairy cow

**DOI:** 10.3389/fvets.2022.1020166

**Published:** 2022-11-04

**Authors:** Kamila Peruchi Fernandes, Amauri Alcindo Alfieri, Gabriela Molinari Darold, Fabiana Marques Boabaid, Alais Maria Dall Agnol, Michele Lunardi

**Affiliations:** ^1^Laboratory of Veterinary Pathology, Universidade de Cuiaba, Cuiaba, Brazil; ^2^Laboratory of Animal Virology, Department of Veterinary Preventive Medicine, Universidade Estadual de Londrina, Londrina, Brazil; ^3^National Institute of Science and Technology for Dairy Production Chain (INCT-LEITE), Universidade Estadual de Londrina, Londrina, Brazil; ^4^Laboratory of Veterinary Microbiology, Universidade de Cuiaba, Cuiaba, Brazil

**Keywords:** cattle, esophagus, rumen, BPV, PCR, sequencing

## Abstract

Bovine papillomavirus (BPV) infection can induce neoplastic lesions in both cutaneous and mucosal epithelia in cattle. This study describes the BPV types associated with proliferative lesions with diverse histopathological features present in the upper alimentary tract of a dairy cow suffering from chronic diarrhea from Midwestern Brazil. At autopsy, warts and plaques composed of multiple spherical nodules were observed in the esophageal mucosa, the areas surrounding and constricting the opening of the cardia, and the rumen pillars. One esophageal papillomatous proliferative lesion and a smooth-surfaced proliferative lesion located at the rumen entrance were evaluated by histopathological and molecular analyses. PCR amplification of partial fragments of the BPV L1 and E1 genes was performed followed by sequencing of the obtained amplicons. Upon histopathological evaluation, the esophageal lesion was classified as a squamous papilloma, whereas the other ruminal proliferative lesion consisted of a fibropapilloma. Direct sequencing of PCR products obtained from ruminal fibropapilloma DNA revealed the presence of BPV2. Sequencing of inserts from selected clones containing partial fragments of the BPV L1 and E1 genes revealed a mixed infection of BPV types 2 and 4 in the esophageal squamous papilloma. The findings reported in our investigation reinforce the association of BPV with benign lesions of the bovine alimentary tract in both single and mixed infections, as previously demonstrated to occur in a buffalo. In addition, this report represents the documentation of the occurrence of massive alimentary papillomatosis associated with BPV types 2 and 4 in cattle raised on lands without infestation by bracken fern in Midwestern Brazil.

## Introduction

Papillomaviruses (PVs) are small, non-enveloped, icosahedral oncogenic viruses, and the PV genome is represented by circular double-stranded DNA of approximately 8,000 base pairs of length. The genomic organization of PVs comprises early (E) and late (L) ORFs, which are classified according to the cell differentiation stage of the epithelium when they are expressed (1). Among all viral proteins encoded by the PV genome, protein E1 recognizes the origin of replication and exhibits intrinsic ATPase/helicase activity, being indispensable for the initiation of viral DNA replication (2). The virally coded protein L1 is the major capsid protein, representing approximately 80% of the total virus protein content. Epitopes that induce the production of neutralizing antibodies are mainly found on the L1 protein (3, 4).

Currently, PVs constitute a widely diverse group of viruses found in many mammalian species as well as in certain birds, reptiles, and fish (5–7). PVs are traditionally designated as viral types. Each viral type represents a complete genome with the L1 gene nucleotide sequence showing at least 10% dissimilarity when compared with the corresponding sequence from any other previously identified PV (8). Bovine papillomavirus (BPV) types are classified into at least five well-established known genera in the *Papillomaviridae* family. BPV types 1, 2, 13, and 14 belong to the *Deltapapillomavirus* genus. BPVs 3, 4, 6, 9, 10, 11, 12, 15, 17, 20, 23, 24, 26, 28, and 29 are grouped in the *Xipapillomavirus* genus. BPV types 5, 8, and 25 are representatives of the *Epsilonpapillomavirus* genus. BPV7 is the only member that represents the *Dyoxipapillomavirus* genus. BPV types 16, 18, and 22 are classified in the *Dyokappapapillomavirus* genus, while BPVs 19, 21, and 27 remain in yet undefined genera (9, 10).

BPV infection can induce neoplastic lesions of both cutaneous and mucosal epithelia with the development of warts observed in a variety of anatomical sites of cattle, such as haired skin, mammary gland, penis, vulva, and upper alimentary mucous membrane. Additionally, BPV type 2 is strongly associated with neoplasia of the urinary bladder, whereas BPV4 is related to upper alimentary tract tumors in cattle. Consumption of bracken fern by BPV-infected cattle is considered an important environmental cofactor in the development of these neoplasms due to the presence of carcinogens, such as ptaquiloside in the plant (11).

This investigation describes the papillomavirus types associated with proliferative lesions with diverse histopathological features present in the upper alimentary tract of a dairy cow suffering from chronic diarrhea from Midwestern Brazil.

## Materials and methods

### Clinical presentation and samples

A Girolando dairy cow aged 8 years old was attended at the Veterinary Teaching Hospital, University of Cuiaba, Mato Grosso, Midwestern Brazil. Its clinical alterations were recorded based on clinical examination and information obtained from the owner. The animal presented with extreme weakness associated with chronic intermittent diarrhea that occurred for several years and alopecic dermatosis. In addition, it was reported that the cow had delayed development compared to other animals in the herd. On physical examination, sole ulcers, increased joint volume, enlargement of the subscapular lymph node, skin with multifocal areas of alopecia, diarrhea, prostration and head turned to the left side were observed. After four days of hospitalization, the animal died.

After natural death, a complete autopsy was performed. At autopsy, fragments of the skin, esophagus, rumen, reticulum, omasum, abomasum, small and large intestines, liver, kidneys, urinary bladder, lungs, heart, spleen, lymph nodes, and brain, as well as samples of papillomatous proliferative lesions from the esophagus and rumen, and fragments of smooth-surfaced proliferative lesions were collected. All tissues were fixed by immersion in 10% neutral buffered formalin and processed for routine histopathological evaluation with hematoxylin and eosin staining. Furthermore, one fragment of the papillomatous proliferative lesion from the esophagus and another fragment of smooth-surfaced proliferative lesion located at the rumen entrance were also collected. Each tissue fragment was maintained at −80 °C until used for the molecular detection of the BPV genes by PCR assays.

Sample collection and all procedures performed in this study were based on ethical and animal welfare considerations.

All applicable international, national, and/or institutional guidelines for the care and use of animals were followed. Written informed consent was obtained from the owner for the participation of his animal in this study.

### DNA extraction from upper alimentary tract lesions

For molecular analyses, frozen fragments of the two selected esophageal and ruminal proliferative lesions were disrupted and homogenized with the aid of TissueLyser LT (Qiagen, Hilden, Germany).

The purification of total DNA from these samples was achieved by using the QIAamp DNA Blood Mini kit (Qiagen, Hilden, Germany) following the manufacturer's instructions. Purified DNA was eluted in 50 μL of ultrapure sterile water and maintained at −20°C until further use in the PCR assays. Aliquots of ultrapure sterile water were included as negative controls in all DNA extraction procedures.

### PCR amplification of partial fragments of L1 and E1 genes

To amplify subgenomic DNA from BPV papillomaviruses that were potentially present in proliferative lesions collected from the upper alimentary tract of the evaluated dairy cow, the modified PCR assay (12) using the primer pair FAP59 (forward: 5′-TAACWGTIGGICAYCCWTATT-3′) and FAP64 (reverse: 5′-CCWATATCWVHCATITCICCATC-3′), which amplifies a partial fragment of approximately 480 bp of L1 gene of PV in general (13), was employed. Additionally, the degenerate primer pair AR-E1F2 (forward: 5′- ATGGTNCAGTGGGCNTATGA-3′) and AR-E1R4 (reverse: 5′- ATTNCCATCHADDGCATTTCT-3′), which was designed to detect a conserved region of approximately 552 bp of the E1 gene, was also tested (14).

PCR mixtures for both PCRs consisted of 2 μL of the extracted DNA and 48 μL of PCR mix, which included 1 μM of each primer, 200 μM of each deoxynucleoside triphosphate (dNTP) (Invitrogen, Carlsbad, USA), 2.5 U of Platinum *Taq* DNA polymerase (Invitrogen, Carlsbad, USA), 1× PCR buffer (20 mM Tris-HCl [pH 8.4] and 50 mM KCl), 1.5 mM MgCl_2_, and ultrapure sterile water to make up the final volume. Amplification was performed using the following cycling profile: an initial step of 5 min at 94°C; followed by 45 cycles of 1 min at 94°C, 1 min at 50°C for primer annealing, and 1 min at 72°C; and a final extension step of 5 min at 72°C. Aliquots from the PCR-amplified products were analyzed by electrophoresis in a 2% agarose gel stained with ethidium bromide (0.5 mg/mL) and examined under UV light.

### Direct sequencing of obtained amplicons

Initially, PCR products representing partial fragments of the L1 and E1 genes of PVs were excised from agarose gels and purified using the Illustra GFX PCR DNA and Gel Band Purification kit (GE Healthcare, Little Chalfont, United Kingdom). Direct sequencing was then performed using the BigDye Terminator v3.1 Cycle Sequencing kit (Applied Biosystems, Carlsbad, USA) with the corresponding forward and reverse primers in a 3,500 Genetic Analyzer (Applied Biosystems, Carlsbad, USA) according to the manufacturer's instructions.

The quality analysis of the chromatogram readings generated was examined with the Phred application. The sequences were accepted if the base quality was ≥ 20. The consensus sequences were determined using CAP3 software, and their identities were compared with all sequences deposited in the GenBank database using the BLASTn program.

### Molecular cloning and sequencing of inserts

Molecular cloning of purified amplicons whose direct sequencing did not generate good-quality nucleotide sequences was performed using the TOPO TA Cloning Kit for Sequencing (Invitrogen, Carlsbad, USA) according to the manufacturer's instructions. Inserts from two selected clones were then sequenced in both directions using the M13 primers.

### GenBank accession numbers

The partial nucleotide sequences of the L1 and E1 genes of BPV types herein identified infecting a fibropapilloma and a squamous papilloma from the upper alimentary tract of a dairy cow were deposited in the GenBank database under the accession numbers MW161163 to MW161168.

### Reverse transcription followed by PCR for bovine viral diarrhea virus testing

An aliquot of total blood was obtained from the animal during the clinical examination at the Veterinary Teaching Hospital for bovine viral diarrhea virus testing. Total RNA was purified from the sample by using TRIzol LS Reagent (Invitrogen, Carlsbad, USA) according to the manufacturer's instructions. Aliquots of ultrapure sterile water and MDBK (Madin Darby bovine kidney) cells infected with the BVDV NADL strain were used as negative and positive controls, respectively.

Briefly, to amplify a partial fragment of the 5' untranslated region (5' UTR) of the BVDV genome, viral RNA was initially converted into cDNA using M-MLV reverse transcriptase (Invitrogen, Carlsbad, USA) with a specific reverse primer. For PCR, the following reaction mixture was used: 5 μL of cDNA, 20 pmol of each primer (324 and 326) (15), 100 μM of each deoxynucleoside triphosphate (dNTP) (Invitrogen, Carlsbad, USA), 2.5 U of Platinum *Taq* DNA polymerase (Invitrogen, Carlsbad, USA), 1× PCR buffer (20 mM Tris-HCl [pH 8.4] and 50 mM KCl), 2.0 mM MgCl_2_, and ultrapure sterile water to make up a final volume of 50 μL. Amplification was performed with the following cycling profile: an initial step of 2 min at 94°C; followed by 40 cycles of 1 min at 94°C, 1 min at 56°C for primer annealing, and 1 min at 72°C; and a final extension step of 7 min at 72°C.

## Results

**Figure 1:**
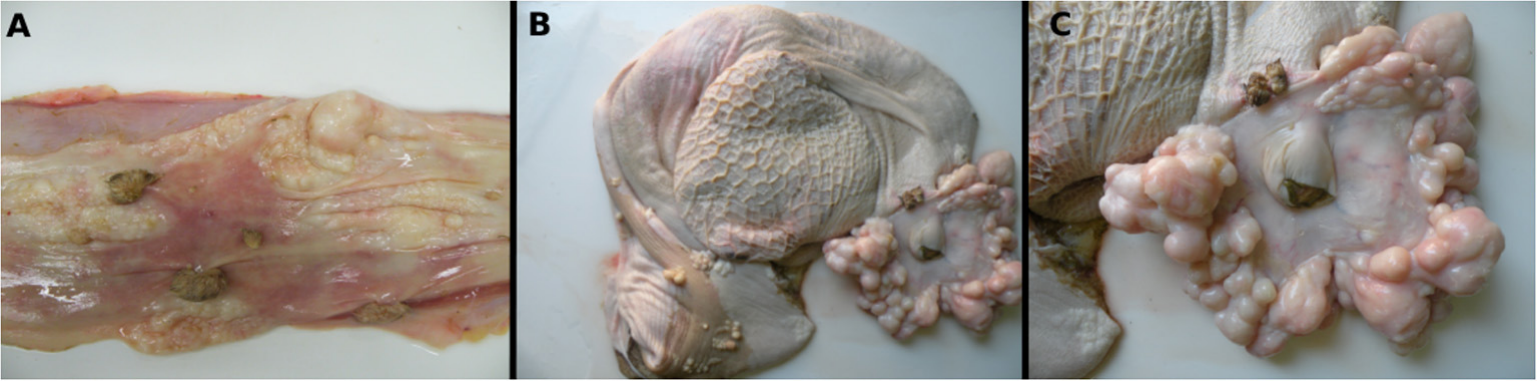
Macroscopic features of a fibropapilloma and papillomas in the esophagus, rumen, and cardia: **(A)** esophageal mucosa, multiple pedunculated nodules with a verrucous cauliflower-like appearance, consistent with papillomas, and others with spherical to plaque-like conformation and smooth surface, characterizing fibropapillomas; **(B)** rumen and reticulum, note in the mucosa of the rumen multifocal spherical and plaque-like nodules with a smooth, shiny white surface, distributed overwhelmingly along the rumen pillars and around the cardia; a single papillomatous nodule can be seen near the cardia; **(C)** rumen, a closer view of the cardia with numerous nodules with a smooth shiny surface and pearly-white aspect are disposed all around the periphery of the cardia. There is also a single papilloma near to the cardia.

At the macroscopic evaluation, alterations observed included poor body condition and slightly pale mucous membranes. The skin had multifocal areas of alopecia, erythema, and nodular exophytic papillomatous lesions with cauliflower-like aspects (papillomas) that were either pedunculated or sessile, ranging from 0.3 to 1.0 cm in diameter. In the abdominal, thoracic cavities, and pericardial sac, a moderate amount of yellowish serous fluid was observed as well as edema of the wall of the digestive tract and lymph nodes. The esophageal mucosa also had multiple white to gray papillomatous nodules with 0.5–1.5 cm in diameter, and pearly-white smooth spherical nodules or plaques that were 0.1–2.0 cm in diameter with a white color on cut surface (Figure 1A). Surrounding and constricting the opening of the cardia numerous smooth masses measuring approximately 0.1–6.0 cm in diameter were seen associated with occasional papillomatous nodules (Figure 1C). Multiple smooth-surfaced nodules were also seen multifocally in the rumen mucosa near to the cardia, and especially on the rumen pillars (Figure 1B).

**Figure 2:**
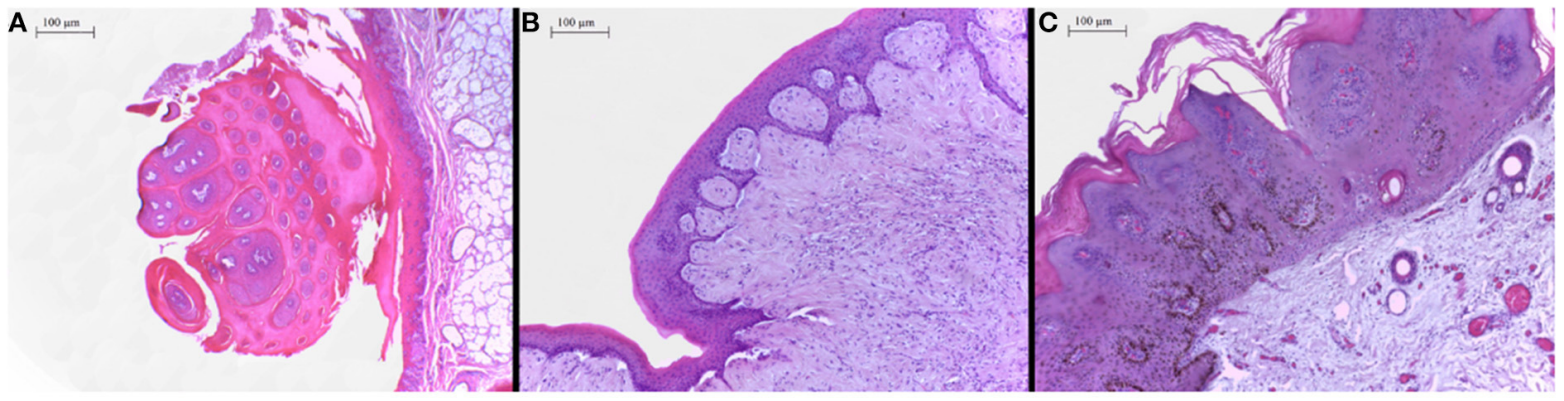
**(A)** Proximal esophagus, exophytic proliferation of papillary projections of stratified squamous epithelium forming thick fronds supported by a thin core of fibrovascular stroma converging to a common peduncle. Hematoxylin-eosin (HE) 4×. **(B)** Ruminal fibropapilloma with neoplastic proliferation of squamous epithelial cells and mesenchymal cells forming streams, expanding the submucosa and elevating the overlying proliferated mucosa. The epithelial component forms numerous acanthomatous projections into the mesenchymal component. Hematoxylin-eosin (HE) 10×. **(C)** Skin, face, a thick proliferation of squamous epithelial cells is seen forming a broad-based well-delimited nodule with moderate hyperkeratosis.

In the histopathological evaluation of the neoplastic lesions with papillomatous aspect located at the esophagus and rumen, a well-differentiated proliferation of stratified squamous epithelial cells was observed. The epithelial cells were disposed in thick layers and formed papilliform projections supported by a thin connective stromal core, occasionally forming a peduncle. The cells exhibited evident desmosomal junctions, occasionally with numerous keratohyalin granules. Into the stratum spinosum of the epithelial proliferation, there were sparse individual or small groups of cells exhibiting cellular degeneration, having a clear cytoplasm and often a condensed nucleus corresponding to koilocytes. These lesions were classified as squamous papillomas (Figure 2A). The smooth-surfaced nodules histologically presented a well-delimited, unencapsulated benign neoplastic proliferation of spindle cells covered by a moderately acanthotic squamous epithelium. The spindle cells were arranged in interlacing streams and bundles supported by moderate collagenous connective stroma. The histological characteristics seen in those nodules are consistent with fibropapillomas (Figure 2B). On the skin, multiple squamous papillomas similar to that of the rumen were also observed (Figure 2C), in addition to multifocal mild lymphoplasmacytic dermatitis, discrete multifocal areas of liquefactive necrosis of the epidermis and moderate hyperkeratosis. The tumoral lesions were classified into squamous papillomas or fibropapillomas according to histopathological characteristics described by Munday et al. (16). There were no other significant histological findings in the remaining tissues.

PCR products, representing partial fragments of the L1 and E1 genes of the BPV genome, were successfully amplified by the selected PCR assays using the FAP59/FAP64 and AR-E1F2/AR-E1R4 primer pairs only in the DNA purified from the ruminal fibropapilloma, generating amplicons with the expected lengths of approximately 480 and 552 bp, respectively. Regarding PCR amplification of DNA extracted from the squamous papilloma located at the esophagus, while PCR assay employing primers for the E1 gene properly yielded an amplicon of the expected molecular size, the PCR method for the L1 gene performed with degenerate primers FAP generated only a faint band of approximately 480 bp, which could not be directly sequenced due to an insufficient amount of DNA. In addition, all negative controls included in both PCR assays were not amplified.

Direct sequencing of both amplicons obtained from the ruminal fibropapilloma DNA generated high-quality nucleotide sequences representing partial fragments of PV L1 and E1 genes belonging to BPV type 2. The highest identity observed at the L1 nucleotide sequence with BPV sequences deposited in GenBank was 99.76% compared with the L1 gene of BPV2 strain 16PL isolated from an equine sarcoid from a horse from Poland (Accession number: KF284153). The nucleotide identity of 100% was shown at the E1 gene with BPV2 strain SW, which was identified from a cutaneous papilloma in a dairy cow from China (Accession number: KC878306).

In contrast, direct sequencing of the PCR product amplified with the primer pair targeting the E1 gene for the esophageal squamous papilloma DNA sample provided only low-quality nucleotide sequences. However, analysis of these low-quality sequences by BLASTn showed that the positive-strand nucleotide sequence presented a higher identity with BPV2, whereas the negative-strand sequence presented a high identity with BPV4 E1 nucleotide sequences. To investigate the occurrence of mixed infection in the squamous papilloma collected from the esophagus of the dairy cow, L1 and E1 amplicons obtained for this lesion were cloned in *E. coli*. Sequencing of inserts from two selected clones from the FAP amplicon exhibited identical sequences and revealed the presence of a partial L1 gene sequence of BPV4 with an identity of 99.06% with the BPV4 reference strain (Accession number: X05817). In contrast, sequencing of inserts purified from two selected clones from the AR-E1F2/E1R4 PCR product demonstrated identification of E1 gene partial sequences of both BPV2 and BPV4, presenting the highest identities of 100 and 99.56% with BPV2 strain SW (Accession number: KC878306) and BPV4 reference strain (Accession number: X05817), respectively.

## Discussion

Through PCR amplification employing two sets of degenerate primers able to amplify a broad range of PV types followed by molecular cloning and sequencing, both BPV types 2 and 4 were demonstrated to infect the upper alimentary tract of a dairy cow from Midwestern Brazil. The mixed infection by the two viral types was characterized in one of the benign lesions examined. The ruminal fibropapilloma had BPV2 identified by direct sequencing of amplicons obtained from E1 and L1 ORFs. In the esophageal lesion classified as squamous papilloma, BPV4 was identified through cloning and sequencing of the L1 gene fragment, whereas both BPV2 and 4 were shown to be coinfecting the same papilloma by sequencing inserts from two clones representing partial fragments of the E1 gene.

The participation of BPV types 2 and 4 in the etiology of tumors from mucous membranes lining the alimentary canal was previously established in initial studies. Although BPV4 has been classically associated with epithelial papillomas and malignancies of the upper gastrointestinal tract of cattle, the participation of BPV2 in the induction of benign lesions has been rarely reported (17–19). Alimentary fibropapillomatosis of cattle, which was characterized by lesions located at the esophagus and rumen, was investigated, and infection by BPV type 2 was revealed. Curiously, attempts to detect mature virus failed, demonstrating that epithelium and underlying fibroblasts of the alimentary tract are nonpermissive to productive viral infection with BPV2. Since BPV2 DNA has not been detected in bovine alimentary cancers, no relationship with malignant transformation has been established (17).

Whereas large amounts of mature viral progeny can be detected in warts of skin and mucosa infected by BPV, it has been documented that attempts to identify mature virions and their antigens had failed in carcinomas of the upper alimentary tract in cattle (20, 21). This fact has been extensively confirmed by detailed surveys of malignant tumors of the alimentary canal, in which BPV4 DNA is rarely detected while replicating virus is always absent (18, 22, 23). These consistent findings have demonstrated that although BPV4 is a common causative agent of alimentary papillomas, its continuous presence and gene expression are not necessary for progression to and maintenance of the malignant transformed state (18).

Therefore, the identification of BPV2 DNA in the fibropapilloma collected from the rumen of the affected cow as well as the presence of BPV4 DNA in the esophageal squamous papilloma examined are consistent with the findings reported by other studies, in which BPV types classified in the *Deltapapillomavirus* genus are known to cause hyperproliferation of the fibroblasts of the underlying dermis, as previously documented for BPV2 causing alimentary fibropapillomas in cattle, whereas BPV4 classified in the *Xipapillomavirus* genus is purely epitheliotropic papillomavirus that exclusively infects the mucus epithelium of the bovine alimentary tract without fibroblast involvement (17–19, 24). In contrast, the presence of BPV2 in a mixed infection with BPV4 in a squamous papilloma of the alimentary canal of cattle was not previously reported to the authors' knowledge.

On the other hand, an etiopathological investigation of the presence of BPV types 1, 2, 5, and 10, by means of specific PCR assays followed by direct sequencing in wart-like lesions collected from the mouth, esophagus, rumen, reticulum, and intestine of both cattle and buffalos from India revealed the presence of only BPV5 DNA in ruminal papillomatosis of cattle. Conversely, BPVs 1, 2, and 5 DNA were identified in benign lesions of the rumen and reticulum of buffalos, being a mixed infection by viral types 1 and 2 characterized in a wart from the reticulum of an affected buffalo (25). Given that BPV type 5 was the most prevalent viral type detected in papillomas from the gastrointestinal tract in the Indian study, its association with papillomatosis of the upper alimentary canal of cattle along with BPV4 and BPV2 seems reasonable. However, employing unspecific molecular tools, such as the strategy employed in our study, to investigate BPV types that might be associated with these lesions from the gastrointestinal tract is highly recommended.

Although BPV-induced papilloma is typically considered self-limiting and spontaneously regresses in immunocompetent individuals, regression is prevented, resulting in the spread and massive persistent papillomatosis in immunosuppressed animals both in cutaneous and mucous epithelia (22, 26). Common causes of chronic immunosuppression in cattle affected by florid papillomatosis are the ingestion of bracken fern and infection by BVDV (22, 27). Given that the cow evaluated in this study had a recurrent and long-term clinical manifestation of diarrhea and was grazing on land where brackens were absent, infection by BVDV was investigated by testing a blood sample through RT-PCR. However, despite testing of a blood sample for BVDV RNA in the period of its permanence at the Veterinary Teaching Hospital, amplification of a partial fragment of the 5' UTR of the BVDV genome was not obtained, whereas the positive control represented by the NADL strain generated a PCR product of the expected size (288 bp). The natural occurrence of extensive alimentary papillomatosis in cattle from diverse geographical regions was previously associated with herds with a history of diagnosis of BVDV infection or exposure to pastures with bracken fern, which ultimately interferes with immune responses to papillomaviral infection (22, 27, 28). Since both factors were absent in the cow investigated in this study, another undetermined cofactor might be present that resulted in immunosuppression leading to persistent and widespread papillomatosis.

In a recent survey conducted in southern Brazil, PCRs using the FAP primer pair and consensus primers for the BPV L1 gene failed to identify BPV DNA in 47 papillomas of the mouth and esophageal mucosa of cattle suffering from squamous cell carcinomas of the upper digestive tract (23). In contrast, the findings shown in our investigation reinforce the association of BPV with benign lesions presenting diverse histopathological features of the bovine alimentary tract, both in single and mixed infections, as previously demonstrated to occur in a buffalo (25). In addition, this report represents the documentation of the occurrence of massive alimentary papillomatosis associated with BPV types 2 and 4 in cattle raised on lands without infestation by bracken fern in Midwestern Brazil.

## Data availability statement

The nucleotide sequences of the L1 and E1 genes of BPV types herein obtained are available in the Genbank database under the accession numbers MW161163 to MW161168.

## Ethics statement

Ethical review and approval were not required because this study involved only one animal naturally affected by papillomatosis that died spontaneously. Its lesions were further evaluated by histopathological and molecular analyses with written informed consent from the animal owner.

## Author contributions

KF: conceptualization, methodology, review, and editing. AA: validation, formal analysis, investigation, resources, review, editing, and funding acquisition. GD, FB, and AD: methodology, review, and editing. ML: conceptualization, validation, formal analysis, investigation, resources, original draft preparation, supervision, and funding acquisition. All authors contributed to the article and approved the submitted version.

## Funding

This work was supported by the National Institute of Science and Technology of Dairy Production Chain (CNPq/INCT-Leite) [Grant No. 465725/2014-7].

## Conflict of interest

The authors declare that the research was conducted in the absence of any commercial or financial relationships that could be construed as a potential conflict of interest.

## Publisher's note

All claims expressed in this article are solely those of the authors and do not necessarily represent those of their affiliated organizations, or those of the publisher, the editors and the reviewers. Any product that may be evaluated in this article, or claim that may be made by its manufacturer, is not guaranteed or endorsed by the publisher.
